# Stability Indicating HPLC Method for Simultaneous Determination of Mephenesin and Diclofenac Diethylamine

**DOI:** 10.4103/0250-474X.51950

**Published:** 2009

**Authors:** S. V. Mulgund, M. S. Phoujdar, S. V. Londhe, P. S. Mallade, T. S. Kulkarni, A. S. Deshpande, K. S. Jain

**Affiliations:** Department of Pharmaceutical Chemistry, Sinhgad College of Pharmacy, Vadgaon (Bk), Pune-411 041, India

**Keywords:** Mephenesin, diclofenac diethylamine, stress testing, degradation products, stability indicating method, HPLC

## Abstract

A simple, specific, accurate and stability-indicating reversed phase high performance liquid chromatographic method was developed for the simultaneous determination of mephenesin and diclofenac diethylamine, using a Spheri-5-RP-18 column and a mobile phase composed of methanol: water (70:30, v/v), pH 3.0 adjusted with *o*-phosphoric acid. The retention times of mephenesin and diclofenac diethylamine were found to be 3.9 min and 14.5 min, respectively. Linearity was established for mephenesin and diclofenac diethylamine in the range of 50-300 μg/ml and 10-60 μg/ml, respectively. The percentage recoveries of mephenesin and diclofenac diethylamine were found to be in the range of 99.06-100.60% and 98.95-99.98%, respectively. Both the drugs were subjected to acid, alkali and neutral hydrolysis, oxidation, dry heat, photolytic and UV degradation. The degradation studies indicated, mephenesin to be susceptible to neutral hydrolysis, while diclofenac diethylamine showed degradation in acid, H_2_O_2_, photolytic and in presence of UV radiation. The degradation products of diclofenac diethylamine in acidic and photolytic conditions were well resolved from the pure drug with significant differences in their retention time values. This method can be successfully employed for simultaneous quantitative analysis of mephenesin and diclofenac diethylamine in bulk drugs and formulations.

Mephenesin (MEP), 3-(2-methylphenoxy)-1,2-propanediol (fig. 1), is a white crystalline powder, almost odorless, slightly soluble in water but freely soluble in alcohol, chloroform and solvent ether. MEP is centrally acting muscle relaxant and a topical analgesic. It is official in Indian Pharmacopoeia[1], which recommends a titrimetric method for its analysis. Diclofenac diethylamine (DDEA), diethylammonium 2-[(2,6-dichloroanilino)phenyl]acetate (fig. 2) is a white to light beige crystalline powder, sparingly soluble in water and acetone, freely soluble in ethanol and methanol. It is commonly used as an analgesic and antiinflammatory agent. DDEA is official in British Pharmacopoeia[2], which recommends HPLC and HPTLC methods for its analysis.

**Fig. 1:**
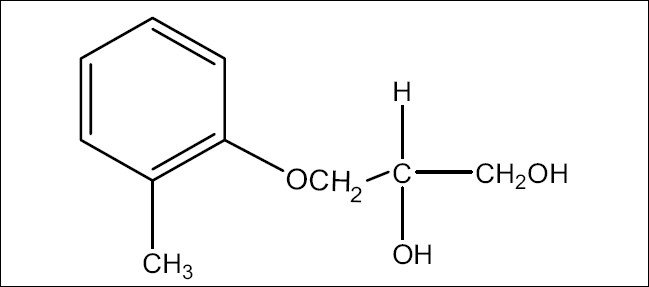
Structure of mephenesin (MEP)

**Fig. 2:**
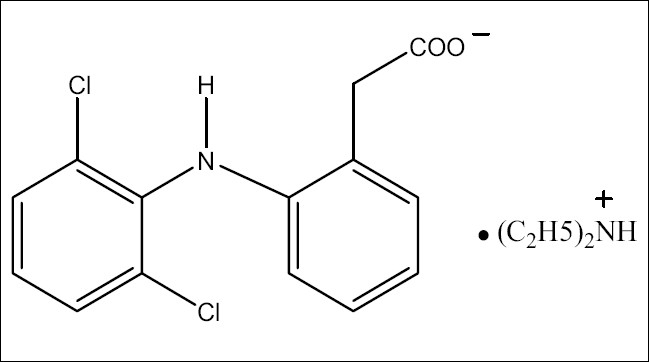
Structure of Diclofenac diethylamine (DDEA)

MEP and DDEA combination gel is a recently introduced topical analgesic anti-inflammatory formulation in Indian market. Literature survey reveals that many analytical methods are reported for determination of MEP[3–6] and DDEA[7–17] individually. However, no method is reported for simultaneous estimation of these two drugs by reverse phase HPLC.

The International Conference on Harmonization (ICH) guideline entitled “Stability testing of new drug substances and products” requires that stress testing be carried out to elucidate the inherent stability characteristics of the active substance[18]. An ideal stability-indicating method is one that resolves the drug and its degradation products efficiently. Consequently, the implementation of an analytical methodology to determine MEP and DDEA simultaneously, in presence of its degradation products is rather a challenge for pharmaceutical analyst. Therefore, it was thought necessary to study the stability of MEP and DDEA under acidic, alkaline, oxidative, UV and photolytic conditions. This paper reports validated stability-indicating HPLC method for simultaneous determination of MEP and DDEA in presence of their degradation products. The proposed method is simple, accurate, reproducible, stability-indicating and suitable for routine determination of MEP and DDEA in combined dosage form. The method was validated in compliance with ICH guidelines[19,20].

## MATERIALS AND METHODS

MEP and DDEA of pharmaceutical grade were kindly supplied as gift samples by Nulife Pharmaceuticals, Pune, India, and were certified to contain 99.65% (w/w) and 99.35% (w/w) respectively, on dried basis. Methanol and water used were of HPLC grade and were purchased from Spectrochem Pvt. Ltd. Mumbai, India. The gel formulation (Systaflam Gel, Systopic Laboratories Pvt. Ltd., Bangalore, India) containing 5% w/w of MEP and 1.16% w/w of DDEA was procured from local market and used for analysis. The liquid chromatographic system was of Perkin Elmer (USA), series 200, which consisted of following components: a binary gradient pump, variable wavelength programmable UV/Vis detector, a manual injection facility with 20 μl fixed loop. The chromatographic analysis was performed using Total ChromNavigator version 6.3 software on a Spheri-5-RP-18 column (250×4.6 mm, 5 μm particle size). In addition, an electronic balance (Shimadzu AX200), a pH meter (Systronics model EQMK VI), a sonicator (Spectra Lab, model UCB 40), a hot air oven (Labhosp), UV chamber (Labhosp) were used in this study.

### Preparation of Mobile Phase and Stock Solutions:

Seven hundred millilitres of methanol and 300 ml of water were mixed and pH of mixture was adjusted to 3.0 with *o-*phosphoric acid. This mixture was sonicated for 10 min and filtered through 0.22 μm membrane filter and used as mobile phase. Stock solutions were prepared by weighing 10 mg each of MEP and DDEA. The weighed drugs were transferred to two separate 10 ml volumetric flasks. Volumes were made upto the mark with methanol to obtain a solution containing 1000 μg/ml of MEP and DDEA. The solutions were further diluted with the same solvent to obtain final concentrations of 100 μg/ml of each drug. The HPLC analysis was performed on reversed-phase high-performance liquid chromatographic system with isocratic elution mode using a mobile phase of methanol:water (70:30, v/v), pH 3.0 adjusted with *o-*phosphoric acid on Spheri-5-RP-18 column (250×4.6 mm, 5 μm particle size) with 1 ml/min flow rate at 221 nm using UV detector.

### Calibration curves for MEP and DDEA:

Gel formulation contained MEP and DDEA in a ratio of 5:1. Appropriate aliquots of MEP and DDEA stock solutions were taken in different 10 ml volumetric flasks and diluted up to the mark with mobile phase to obtain final concentrations of 50-300 μg/ml and 10-60 μg/ml of MEP and DDEA, respectively. The solutions were injected using a 20 μl fixed loop system and chromatograms were recorded. Calibration curves were constructed by plotting average peak areas *versus* concentrations and regression equations were computed for both the drugs (Table 1).

**TABLE 1:** LINEAR REGRESSION DATA FOR CALIBRATION CURVES

Parameters (Units)	MEP	DDEA
Linearity range (μg/ml)	50-300	10-60
r^2^±SD	0.9994±0.00038	0.9988±0.0085
Slope±SD	0.35198±0.0104	0.72821±0.053
Intercept±SD	0.898333±0.08411	0.38333±0.4876
Average of SE of estimation	1.182073	0.721271

MEP is mephenesin, DDEA is diclofenac diethyl amine, SE is the standard error of the mean, SD is standard deviation for n = 3 observations.

### Analysis of Marketed Formulation:

About 1000 mg of gel containing 50 mg of MEP and 11.6 mg of DDEA was accurately weighed and transferred into a 100 ml volumetric flask containing 50 ml methanol, sonicated until the gel got dissolved and diluted upto the mark with same solvent to get final concentrations of 500 μg/ml and 116 μg/ml of MEP and DDEA, respectively. The above solution was filtered using Whatman filter paper No 1. Appropriate volume of the aliquot was transferred to a 10 ml volumetric flask and the volume was made upto the mark with mobile phase to obtain a solution containing 50 μg/ml of MEP and 11.6 μg/ml of DDEA. A 20 μl volume of above sample solution was injected into HPLC and peak areas were measured under optimized chromatographic conditions.

### Method Validation:

The method of analysis was validated as per the recommendations of ICH[21] and USP[22] for the parameters like accuracy, linearity, precision, detection limit, quantitation limit and robustness. The accuracy of the method was determined by calculating percentage recovery of MEP and DDEA. For both the drugs, recovery studies were carried out by applying the method to drug sample to which known amount of MEP and DDEA corresponding to 80, 100 and 120% of label claim had been added (standard addition method). At each level of the amount six determinations were performed and the results obtained were compared.

Intraday and interday precision study of MEP and DDEA was carried out by estimating the corresponding responses 3 times on the same day and on 3 different days for the concentration of 50 μg/ml and 10 μg/ml of MEP and DDEA, respectively. The limit of detection (LOD) and limit of quantitation (LOQ) were calculated using following formulae: LOD= 3.3(SD)/S and LOQ= 10 (SD)/S, where SD=standard deviation of response (peak area) and S= average of the slope of the calibration curve.

System suitability tests are an integral part of any chromatographic analysis method which are used to verify reproducibility of the chromatographic system. To ascertain its effectiveness, certain system suitability test parameters were checked by repetitively injecting the drug solution at the concentration level 50 μg/ml and 10 μg/ml for MEP and DDEA, respectively to check the reproducibility of the system and the results are shown in Table 2.

**TABLE 2:** SUMMARY OF VALIDATION AND SST PARAMETERS

Parameter (Units)	MEP	DDEA
Linearity range (μg/ml)	50-300	10-60
Correlation coefficient	0.9994±0.00038	0.9988±0.0085
LOD (μg/ml)	0.20	0.25
LOQ (μg/ml)	0.50	0.45
Recovery (%)	100.04	99.46
Precision (%RSD)		
Interday (n=3)	1.5	0.44
Intraday (n=3)	1.9	0.49
Robustness	Robust	Robust
Retention Time±allowable time (min.)	3.9±0.2	14.5±0.2
Resolution	2.335	1.463
Theoretical Plates	4500	2300
Tailing Factor (asymmetry factor)	1.02	1.3

SST stands for system suitability test.

For robustness evaluation of HPLC method a few parameters like flow rate, percentage of methanol in the mobile phase and pH of mobile phase were deliberately changed. One factor was changed at one time to estimate the effect. Each factor selected was changed at three levels (−1, 0, +1) with respect to optimized parameters. Robustness of the method was done at the concentration levels 50 μg/ml and 10 μg/ml for MEP and DDEA, respectively and the results are shown in Table 3.

**TABLE 3:** ROBUSTNESS EVALUATION OF METHOD FOR MEP AND DEEA

Factor	Level^a^	MEP T_r_^b^	DDEA T_r_^b^
A: Flow Rate (ml/min)			
0.9	−1	3.7	14.6
1.0	0	3.9	14.8
1.1	+1	4.0	14.9
Mean±SD		3.86±0.15	14.7±0.15
B:Percentage of methanol in the mobile phase (v/v)			
69	−1	3.8	14.5
70	0	3.9	14.8
71	+1	4.1	15.1
Mean±SD		3.93±0.15	14.8±0.3
C: pH of mobile phase			
2.9	−1	3.5	14.5
3.0	0	3.9	14.8
3.1	+1	4.3	15.2
Mean ±SD		3.9±0.4	14.83±0.35

Concentrations level used for robustness evaluation was 50 μg/ml

a Three factors were slightly changed at three levels (−1, 0, 1) and

b retention time.

### Forced degradation studies:

Forced degradation studies of both the drugs were carried out under conditions of hydrolysis, dry heat, oxidation, UV light and photolysis. MEP and DDEA were weighed (100 mg each) and transferred into two 50 ml volumetric flasks and diluted up to the mark with methanol to give 2000 μg/ml concentration of each drug. These stock solutions were used for forced degradation studies.

Forced degradation in basic media was performed by taking 10 ml of stock solution of MEP and DDEA each in separate round bottom flasks. Then 10 ml of 5 N NaOH was added and these mixtures were heated for upto 8 h at 70° in dark, in order to exclude the possible degradative effect of light. Forced degradation in acidic media was performed by keeping the drug in contact with 1N HCl for upto 30 h at ambient temperature as well as heating for up to 8 h at 70° in dark. Degradation with hydrogen peroxide was performed by taking 10 ml of stock solution of MEP and DDEA in two different flasks and adding 10 ml of 30% (w/v) hydrogen peroxide in each of the flasks. These mixtures were kept for upto 4 days in the dark. To study neutral degradation, 10 ml of stock solutions of MEP and DDEA taken in two different flasks, then 10 ml of HPLC grade water was added in each flask and these mixtures were heated for 6 h at 70° in the dark. For dry heat degradation, solid drugs were kept in Petri dish in oven at 100° for 12 h. Thereafter, 10 mg each of MEP and DDEA were weighed and transferred to two separate 10 ml volumetric flasks and diluted up to the mark with methanol. The photostability was also studied by exposing above stock solutions (1000 μg/ml) of both the drugs to direct sunlight in summer days for 5 h on a wooden plank. For UV degradation study, the stock solutions of both drugs (1000 μg/ml) were exposed to UV radiation of a wavelength of 256 nm and of 1.4 flux intensity for 12 h in UV chamber.

For HPLC analysis, all the degraded sample solutions were diluted with mobile phase to obtain final concentration of 30 μg/ml of MEP and DDEA. Similarly mixture of both drugs in a concentration of 30 μg/ml of MEP and DDEA each was prepared prior to analysis by HPLC. Besides, solutions containing 30 μg/ml of each drug separately were also prepared without performing the degradation of both the drugs. Then 20 μl solution of above solutions were injected into the HPLC system and analyzed under the chromatographic analysis condition described earlier.

## RESULTS AND DISCUSSION

**Fig. 3:**
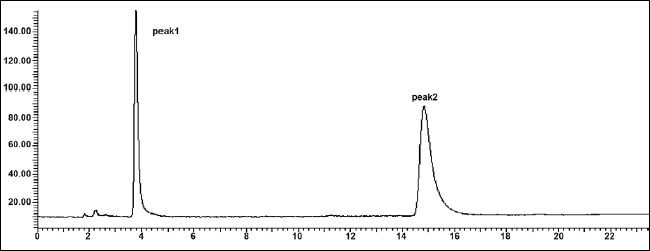
Chromatogram of mixture of MEP and DDEA Mephenesin (MEP, peak 1) with t_R_ of 3.9 min and diclofenac diethylamine (DDEA, peak 2) with t_R_ of 14.5 min.

**Fig. 4:**
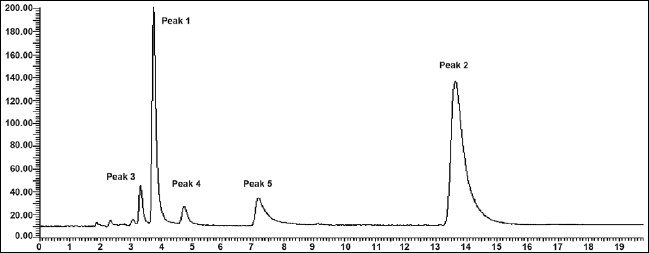
Chromatogram of market formulation of MEP and DDAE Mephenesin (MEP, peak 1) with t_R_ of 3.9 min and diclofenac diethylamine (DDEA, peak 2) with t_R_ of 14.5 min resolved form other excipients (peaks 3, 4 and 5) with t_R_ of 3.5, 4.8 and 7.4 min, respectively.

The mobile phase consisting of methanol: water (70:30, v/v) having pH 3.0 adjusted with *o*-phosphoric acid, at 1ml/min flow rate was optimised which gave two sharp, well-resolved peaks with minimum tailing factor for MEP and DDEA (fig. 3). The retention times for MEP and DDEA were 3.9 min and 14.5 min, respectively. UV overlain spectra of both MEP and DDEA showed that both drugs absorbed appreciably at 221 nm, so this wavelength was selected as the detection wavelength. The calibration curve for MEP and DDEA was found to be linear over the range of 50-300 μg/ml and 10-60 μg/ml, respectively. The data of regression analysis of the calibration curves is shown in Table 1. The proposed method was successfully applied to the determination of MEP and DDEA in their combined gel dosage form. The results for the combination were comparable with the corresponding labelled amounts. The developed method was also found to be specific, since it was able to separate other excipients present in gel from the two drugs (fig. 4).

The LOD for MEP and DDEA were found to be 0.20 μg/ml and 0.25 μg/ml, respectively, while LOQ were 0.50 μg/ml and 0.45 μg/ml, respectively. The results for validation and system suitability test parameters are summarized in Table 2. Results for robustness evaluation for both the drugs are presented in Table 3. Insignificant differences in peak areas and less variability in retention times were observed.

**Fig. 5:**
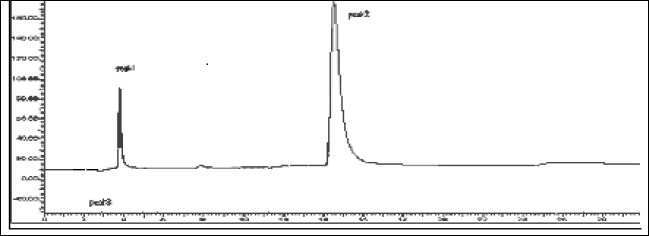
Chromatogram of mixture of MEP and DDEA degraded with neutral hydrolysis Mephenesin (MEP, peak 1) with t_R_ of 3.9 min shows decrease in peak area, but no additional degradation product is observed, diclofenac diethylamine (DDEA, peak 2) with t_R_ of 14.5 min

The degradation study indicated that MEP was susceptible to neutral hydrolysis while it was stable to acid, base, H_2_O_2_, direct sunlight, UV radiation and dry heat under experimental conditions. In neutral hydrolysis the drug degrades as observed by the decreased area in the peak of the drug when compared with peak area of the same concentration of the nondegraded drug, without giving any additional degradation peaks (fig. 5).

**Fig. 6:**
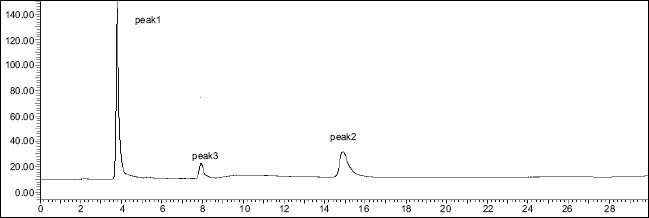
Chromatogram of mixture of MEP and DDEA degraded under acidic conditions Mephenesin (MEP, peak 1) with t_R_ of 3.9 min and diclofenac diethylamine (DDEA, peak 2) with t_R_ of 14.5 min and degradation product of DDEA (peak 3) with a t_R_ of 7.9 min

**Fig. 7:**
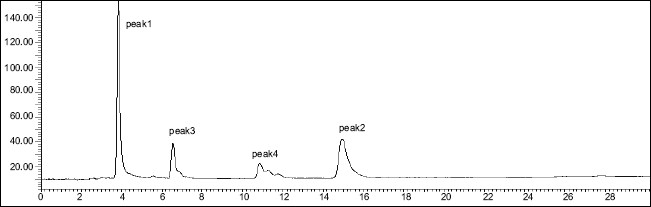
Chromatogram of mixture of MEP and DDEA exposed to direct sunlight Mephenesin (MEP, peak 1) with t_R_ of 3.9 min and diclofenac diethylamine (DDEA, peak 2) with t_R_ of 14.5 min and degradation products of DDEA (peaks 3 and 4) with t_R_ 6.3 min and 10.8 min, respectively

DDEA was found to be susceptible to acid, H_2_O_2_, direct sunlight and UV radiation with maximum degradation under acidic and photolytic conditions; however it showed stability towards alkaline and neutral hydrolysis as well as dry heat degradation. DDEA got degraded into one or two degradation products in the stress conditions of acid hydrolysis as well as photolytic exposure, while both the drugs showed no degradation at 0 h in all the degradation conditions. The chromatogram of the acid degraded sample of DDEA showed one additional peak at t_R_ 7.9 (fig. 6) and chromatogram of photo induced degraded sample showed two additional peaks at t_R_ 6.3 and 10.8 min, respectively (fig. 7) In oxidative and UV degradation, the drug degrades as shown by the decreased areas in the peaks when compared with peak areas of the same concentration of the nondegraded drug, without giving any additional degradation peaks. Percent degradation was calculated by comparing the areas of the degraded peaks in each degradation condition with the corresponding areas of the peaks of both the drugs under non degradation condition. Summary of degradation studies of both the drugs is given in Table 4.

**TABLE 4:** SUMMARY OF DEGRADATION STUDIES FOR MEP AND DDEA

Degradation condition	Time (h/day)	% Degradation		t_R_ (min) of degradation products	
			
		MEP	DDEA	MEP	DDEA
Base, 5 N NaOH (heated, at 70°)	8 h	ND	ND	-----	-----
Acid, 1N HCl (ambient, 30 h and heated, 8 h at 70°)	30 and 8 h	ND	62.1%	----	7.9 min
Oxidative, 30% w/v H_2_O_2_ (ambient, in dark)	4 days	ND	42%	----	**
Neutral hydrolysis (heated, at 70°)	6 h	49.5	ND	**	…….
Dry Heat (100°)	12 h	ND	ND	----	…….
Direct sunlight (photolysis)	5 h	ND	65.5%	….	a. 6.3 min
					b.10.3 min
UV Radiation at 256 nm	12 h	ND	33%	----	**

MEP is mephenesin, DDEA is diclofenac diethylamine, t_R_ stands for retention time, ND represents no degradation observed.

** Represents that no rise of additional degradation peak was observed.

In this reported study, a stability-indicating HPLC method was developed for the simultaneous determination of MEP and DDEA and validated as per ICH guidelines. Statistical analysis proved that the method developed was accurate, precise, and repeatable. The developed method was found to be simple, sensitive and selective for analysis of MEP and DDEA in combination without any interference from the excipients. The method was successfully used for determination of drugs in a pharmaceutical formulation. Assay results for combined dosage form using proposed method showed 99.06±1.40% of MEP and 98.95±0.66% of DDEA. The results indicated the suitability of the method to study stability of MEP and DDEA under various forced degradation conditions *viz.* acid, base, dry heat, neutral, photolytic and UV degradation. It can be concluded that as the method could separate the drugs from their degradation products; it may be employed for analysis of stability samples of MEP and DDEA. However characterization of degradation products was not carried out.
